# An Independent and Public Enquiry Called for

**Published:** 1913-03-15

**Authors:** J. Gordon Macqueen, W. Moir Shepherd

**Affiliations:** Late Resident Medical Officers, York County Hospital.; Late Resident Medical Officers, York County Hospital.


### THE YORK COUNTY HOSPITAL.

### AN INDEPENDENT AND PUBLIC ENQUIRY CALLED FOR.

*To the Editor of* The Hospital.

Sir,—In The Hospital of January 27, 1912, you published a report on the "York County Hospital" throwing some light on its internal administration and management.

On January 13, 1913, we—then resident medical officers in that institution—wrote you confirming your report, and now we wish to put you in possession of details and facts in the hope that something may be done to remedy the evils existent in this public institution.

### Violence and Mischarting.

On October 14, 1912, we made reports to the Medical Board regarding some of the existing conditions. The reports were to this effect:—

That a child in the Children's Ward was discovered by us with suspicious marks upon it, which we attributed to violence. Inquiries from the matron and nurses brought no satisfactory explanation of the marks.

Also in the same ward the temperature of a case had been wilfully mischarted . . . . . . Complaint had been made by us to the matron previously about similar occurrences in the same ward, and no notice had been taken of it.

Also that in many cases there were no records of pulse or respirations, even in a case of osteomyelitis (in this case there was no record for over seven weeks).

### Night Staff Inadequate.

That in our opinion the night-staff of the whole hospital was inadequate for the treatment of the patients; besides there being no emergency night-staff to take theatre or special duty if required. In support of this inadequacy two cases were quoted:—

1. In one ward there was a surgical case which required a great deal of attention. While the ward nurse was attending to this case, a patient in the other division of the ward got up, slipped on the floor and broke his leg.

2. In another ward there was a medical patient who twice fell out of bed. There was one nurse who was doing special duty between these two cases, and in each the "part duty" resulted in the above. The only result of this report was that the obvious parts of it, which could not be got over, were dealt with—viz. the parts relating to the marks upon the child and to the mischarting of temperatures.

As nothing further was done we, on December 27, 1912, sent a report to the House Committee, of which report the following is an extract:—

### An Absence of Control.

"Ever since the time of our separate appointments we have been more than dissatisfied with the general administration and supervision of matters in this house. Some months ago it was reported to the matron that the condition of affairs in Victoria [Children's] Ward was extremely unsatisfactory, that the sister of the ward was . . . . . . failing in her duties as a nurse; but the matron, instead of taking such report from a resident as a matron should, wished to argue the matter and deliberately shut her eyes to the delinquencies involved. The sister was afterwards caught redhanded, and on this being reported to the matron, the latter was exceedingly insolent. (For further details of this we refer you to the Medical Board before whom the case came.) This is a concrete instance, but there are many other derelictions of duty which exist and are not so easily brought home.

A Forecast and Warning.

"In addition . . . . . . the matron did not work in conjunction with the residents, and here we may well refer you to the report of Sir Henry Burdett, who writes in The Hospital of January 27, 1912: 'The impression we gained from a thorough inspection of the hospital [York] was that the internal administration requires modernising from top to bottom; there seems to be too little direct touch from the centre with the actual duties of each sister, nurse, and probationer. Indeed, we gather that the efficiency of the management would be materially increased by the introduction of a controlling hand backed by up-to-date knowledge,' *et seq.*

"In the face of such public strictures of the administration of this institution we fail to understand how you allow the present painful inefficiency to continue.

"Unless radical changes along these suggested lines are promptly made we, the undersigned, cannot see otherwise than to tender you our resignations, separately and conjointly.

"We have struggled hard to raise the efficiency and good name of the hospital during our short tenure of office, but without aid such things are impossible. Any promise of this ancient institution's future usefulness can, in our opinion, never be fulfilled under such conditions as exist at present."

A Final Appeal to the Committee.

As no notice was taken of this report we, on January 11, 1913, sent the House Committee the following letter:—

"We, the resident medical officers, think it right to draw the attention of the House Committee, to certain matters which have occurred during our residence in this hospital. We do this with the sole object of preventing similar occurrences in the future, and in the hope that some action may be taken to improve the present unsatisfactory condition.

"We have already reported to the Medical Board (on October 14, 1912) upon certain matters which we thought demanded serious consideration, and we now beg to bring before you the following cases, which still further indicate the necessity of some reorganisation in the internal arrangements of the hospital.

Patient When Alive Removed to Mortuary.

"1. On September 22, 1912, a patient named Collier was discovered about 11.30 a.m. by the House Physician in the mortuary whilst still alive. A ticket pinned upon the shroud had written upon it that the patient died at 7.30 a.m., and yet at 7.50 a.m. the House Physician was asked concerning the case and gave his orders thereanent; which orders, he afterwards found, were not carried out; nor was he informed of the 'death' until after the patient had been removed to the mortuary.

"Upon the discovery of this fact we decided to remove the patient to the Isolation Ward and informed the matron of our decision, but were met with the astounding reply, 'What advantage is there in having him removed now?'

"The patient ultimately died in the Isolation Ward at 4.45 p.m.

Bed-sore and Suspicious Marks.

"2. On July 6, 1912, at 12.5 a.m. the matron reported to the House Physician (present House Surgeon) about a child in Victoria Ward having a bedsore. This had been present for twelve days, and had been treated without the knowledge of the resident. Arrangements had been made for the child to go out that day.

"3. On October 13, 1912, it was discovered by the House Physician that a child in Victoria Ward had suspicious marks upon it, which we attributed to violence. The facts of the case only came to light with great difficulty and after much unpleasantness.

"There is something radically wrong with a hospital where sisters and nurses . . . . . . fail in their duty to report to the doctor, and still more in their duty to the general public whose welfare they are supposed to guard.

"We trust that in bringing these cases before the notice of the Committee our motive will not be misunderstood. Our responsibilities in connection with the hospital will shortly cease, and its future administration and discipline does not therefore directly concern us; but we feel that we owe it to our successors in office and to the public to inform you of circumstances which, if published, would do infinite harm to the institution, and which must indicate to laymen, as well as to medical men, that the administration of the hospital is very far from being satisfactory.

Neglect of Patients.

"In addition to the specific cases mentioned in our letter to the House Committee dated January 11, 1913, Nos. 1, 2 and 3, we may call attention to the following further cases—viz.:—

"4. Treatment of surgical shock.—A patient who had had a prolonged abdominal operation was taken to a cold ward where the fire had been newly lit, where there was only one hot-water bottle in the bed; and was left, still under the influence of the anæsthetic, upon the trolley, with no one to look after her. The patient died the same afternoon.

"5. A patient admitted after an accident (suffering from slight concussion and shock, and who had an exceedingly bad pulse—not in any fit condition to be moved or washed) was found in the process of being washed ten minutes after admission.

"6. A case taken to the Secretary's office without slippers on her feet.

"7. A case of diphtheria on which tracheotomy had been performed and which required special nursing. A nurse was sent as "special" who had never seen a tracheotomy before, while one who had had a fever training and nursed many similar cases was going about that night doing little or nothing. Result: Membrane was allowed to accumulate in the tube, the tube was coughed out of the wound, and the nurse was unable to replace it. We were urgently called to the case several times because of this, and in the end a second tracheotomy (low) had to be performed.

"8. A nurse who had only had four months' training was left in charge of the Children's Ward at night.

### Children Tied Down in Bed.

"We had also to complain of the following matters:—

"9. Delay (sometimes as much as two hours) in taking a patient to the theatre after instructions had been given for the case to be in readiness at a certain time.

"10. General laxity in carrying out orders.

"11. Neglect to take pulse and respirations already referred to in Children's Ward, but also found in other wards).

"12. Frequent failure to report deaths to the resident in charge.

"13. It was a frequent occurrence to pay a ward visit and never see a sister or nurse, until the visit was almost finished—sometimes not at all.

"14. There was a general lack of ward etiquette.

"15. It seemed to be customary to "lay-out" bodies and remove them to the mortuary as soon as possible.

"16. Frequently external nurses had to be obtained as the hospital staff was so inadequate.

"17. When we first went to the hospital we found that it was customary to tie the children down in bed, no matter what the case might be. The method of tying was by bandage round the shoulders, wrists, and ankles."

### Food and Accommodation Faulty.

Regarding our personal treatment in the said hospital; our living apartments were in our opinion in an extremely unsatisfactory condition. The food was always exceedingly monotonous, on many occasions unpalatable, and several times uneatable. The dishes which we got with our food were always cracked. We complained of this to the Secretary and the matron several times, but no change was made. At last we said that if a change was not made we would break them. On one occasion when all the dishes were cracked, except two plates, we did break them. This was reported to the House Committee by the matron, and regarding this the following is an extract from the letter of December 27, which we sent to the House Committee:—

"Dealing with the question of our personal grievance, we complained to you of the condition of our laundry, but we have not as yet been officially informed of any action taken by you, though we are given to understand it was merely referred to the matron. There is no visible change, it being sometimes worse than it ever was before. Accordingly, as so little notice was taken by you of our last complaint we referred the matter of cracked dishes in this instance to the matron to whom we made personal complaint.

"The food also could do well with a little supervision. It is exceedingly monotonous to come from the operating theatre to meals so lacking in variety!

"The condition of our living apartments too is extremely unsatisfactory, and in support of this we again refer you to the same report by Sir Henry Burdett.

"'We would once more call attention to the rooms of the resident medical officers, which might surely be made more comfortable.'"

"This statement by such a man speaks for itself; nothing further need be added by us."

### Retirement of Residents.

In reply we got the following letter from the Secretary on December 31, 1912:—

"I am directed by the House Committee to send you the following copy of a resolution passed at a meeting held this morning:—

"'Resolved:

"'That in accordance with the resolution adopted on the 24th instant, and confirmed at this present meeting, the two resident medical officers be given one month's notice from to-day to terminate their engagements and the leave of absence granted at the meeting held on the 10th instant be and is hereby cancelled.'"

In return we sent them the following letter on January 7, 1913:—

"We are in receipt of the communication of December 31, 1912, from the House Committee, and beg to state that we cannot accept the resolution therein stated as passed by them. Our refusal is for two reasons, viz:—

"1. That, on December 27, 1912, we wrote you a letter in which we resigned our posts as resident officers of this institution; our resignations therefore dating from that date.

"2. That, even though our resignations had not been handed in, such dismissal by the House Committee is not in accordance with the rules which were in force at the time of our appointment. The rule to which we refer is this, viz.:—

"'The House Surgeon and the House Physician shall be appointed and be removable by the House Committee and members of the Honorary Medical and Surgical Staffs.'"

"As we were appointed by the combined board of House Committee and Medical Board, our dismissal must also come from the combined board.

### A Want of Consideration.

"We cannot restrain ourselves from expressing the opinion that in cancelling our holidays you adopted a very 'dog-in-the-manger' attitude. The House Physician was in ill-health during the week prior to Christmas and was advised by one of the honorary staff to go away for a holiday after the illness, but, as the work at Christmas time was so heavy, he decided to stay on and 'see it through,' in the knowledge that he would be having a holiday so soon after, but this is how he is treated. The House Surgeon did double duty whilst his colleague was in ill-health, working practically night and day, and this is his reward.

"If we took premature action in breaking the cracked dishes before acquainting the House Committee, we regret that action, but we maintain that they should not have been put before us, and we do not retract one iota from the statements made in our letters concerning the hospital and its internal affairs.

"If you as a House Committee wish to see your hospital prosper it is time you gave those internal affairs your most serious consideration."

The only answer which we received to this letter and to that of January 11, 1913, was the following letter from the Secretary, dated January 14, 1913:—

"I am directed by the House Committee to acknowledge the receipt of the letters of the 7th and 11th instant signed by yourself and Dr. S——, which were read at the meeting held this morning."

### The Day Before Departure.

We terminated our engagements at York County Hospital on January 23, 1913.

On the day prior to the appointment of new residents one of us asked a member of the honorary staff for a testimonial. He said that he would consider it. On the same evening a second member was also asked for a testimonial. He said that it had been suggested that we should get testimonials if we would promise not to say anything to the candidates who were coming the following day regarding the condition of affairs existent in the place. On the following morning the member of the honorary staff first referred to came to hospital and informed one of us that he had considered the matter and that he would grant a very good testimonial if an undertaking was given him that we should say nothing about the place to the candidates who were coming that day. This appeared to us to show that these testimonials were to be granted in the light of "hush-money," and they were refused. Both men admitted that our work both medically and surgically had been extremely satisfactory.

### The Treatment of the Nursing Staff.

Regarding the treatment of the nursing staff, there seemed to be a general hesitancy on the part of the nurses themselves to report if they were ill. From an onlooker's standpoint, the only reason which we could assign for such extraordinary conduct was the ordeal (to them) which they had to undergo when they reported themselves.

When they did report themselves there was in several cases a failure . . . . . . to report immediately to the medical staff, and the sick nurses were often left lying in the nurses' home without a fire in their bedrooms—even in December and January. Illnesses too frequently were made light of by the matron and sisters, and the nurses seemed to object to being placed in certain wards on account of the treatment meted out to them by sisters.

One or two specific instances may be of use:—

1. A case of diphtheria, where the patient had been in the nurses' home for several days prior to being seen by the resident or antitoxin administered.

2. A night nurse, who was subject to heart attacks and who had complained during the day, was found by us, on duty at night, cyanosed and gasping for breath, in no fit condition to be even sitting up. Although ordered immediately off duty, she had to remain two hours longer until a day nurse had had a sufficiency of sleep to take her place. This substitute was a nurse who had only had four months' training, and who therefore was in our opinion incompetent of taking charge of a Children's Ward in which there was a case of typhoid, also of pneumonia, empyema, chorea, a bottle-fed baby, and various other medical and surgical cases.

3. A case of sinus suppuration and threatened frontal trouble was left in her bedroom in the month of December without a fire, and seemingly no thought of it, until ordered.

4. A case of severe abdominal colic first felt by the nurse on a Sunday. She reported herself to the sister on Monday: no notice was taken. She reported herself again, to the matron, on Wednesday and was not seen by a doctor until Thursday. At table this nurse had been noticed to have taken little or no food and had asked one evening to be excused from attending the meal.

5. In one of the wards a sister, losing control of herself, threw a book at a nurse in front of the patients.

6. In several instances responsibility which ought to rest with a sister (in cases of mistakes and otherwise) was attempted to be put upon nurses.

A glance over but a few of the facts placed before you must be more than sufficient to convince you of the unhappy position in which the nurses found themselves, self-condemned to a so-called training of three years. Is it any wonder, therefore, that some of them going off on holiday forget to return—one, we believe, even paying (while on holiday) one month's salary in lieu of notice?

### An Intolerable Position.

Imagine for yourself what the nursing of patients could be under such conditions. And we, the unfortunate residents, seeing all these things happening before our eyes, and receiving the shock of finding a live man in the mortuary, felt heavily the daily increasing worry, anxiety, and responsibility which thus fell to our lot.

As neither the Medical Board nor the House Committee of York County Hospital would take notice of our reports or even attempt to aid us in remedying the existent and so apparent evils, we have felt constrained to place before you these details and facts in the hope that you may be able to do something in the interests of the charitable poor of York, of those who give their daughters to nurse those poor, and of the subscribers.

We are, Sir, yours faithfully,

J. Gordon Macqueen, } Late Resident Medical

W. Moir Shepherd, } Officers, York County Hospital.

March 7, 1913.

